# The integration of AI in nursing: addressing current applications, challenges, and future directions

**DOI:** 10.3389/fmed.2025.1545420

**Published:** 2025-02-11

**Authors:** Qiuying Wei, Songcheng Pan, Xiaoyu Liu, Mei Hong, Chunying Nong, Weiqi Zhang

**Affiliations:** ^1^Anesthesia Surgery Center, The First Affiliated Hospital of Guangxi Medical University, Naning, Guangxi, China; ^2^Guangdong Lingnan Nightingale Nursing Academy, Guangzhou, Guangdong, China

**Keywords:** nursing, artificial intelligence, healthcare, nursing practice, integration

## Abstract

Artificial intelligence is increasingly influencing healthcare, providing transformative opportunities and challenges for nursing practice. This review critically evaluates the integration of AI in nursing, focusing on its current applications, limitations, and areas that require further investigation. A comprehensive analysis of recent studies highlights the use of AI in clinical decision support systems, patient monitoring, and nursing education. However, several barriers to successful implementation are identified, including technical constraints, ethical dilemmas, and the need for workforce adaptation. Significant gaps in the literature are also evident, such as the limited development of nursing-specific AI tools, insufficient long-term impact assessments, and the absence of comprehensive ethical frameworks tailored to nursing contexts. The potential of AI to reshape personalized care, advance robotics in nursing, and address global health challenges is explored in depth. This review integrates existing knowledge and identifies critical areas for future research, emphasizing the necessity of aligning AI advancements with the specific needs of nursing. Addressing these gaps is essential to fully harness AI's potential while reducing associated risks, ultimately enhancing nursing practice and improving patient outcomes.

## 1 Introduction

The integration of artificial intelligence into healthcare is progressing rapidly, providing significant potential to transform patient care, clinical decision-making, and healthcare management (1, 98). Within this evolving context, nursing holds a significant role, prepared to influence and benefit from artificial intelligence applications in healthcare delivery.

Artificial intelligence (AI), defined as the replication of human cognitive processes through machines, encompasses technologies such as machine learning, natural language processing, and robotics (2). In nursing, these technologies hold the potential to support various aspects of practice, including patient assessment, care planning, education, and administrative activities (3). Nursing-specific AI tools are designed to address the unique aspects of nursing practice, focusing on functionalities such as patient education, care coordination, and holistic assessments. Unlike general AI applications, these tools are tailored to enhance nursing workflows and improve patient outcomes. As healthcare systems face increasing challenges, such as growing patient demands, complex care needs, and resource constraints, AI offers promising solutions to improve efficiency, accuracy, and patient outcomes (4). The COVID-19 pandemic has further accelerated the adoption of digital health technologies, highlighting both the opportunities presented by AI and the need for nursing professionals to actively engage with these innovations (5).

Despite its potential, integrating AI into nursing presents significant challenges. Ethical concerns, issues surrounding data privacy, and the importance of maintaining human-centered care represent key obstacles (6, 7). In addition, the rapid pace of technological advancements often surpasses the development of regulatory policies and educational frameworks, creating a gap between AI's capabilities and its practical implementation in nursing settings (8).

Previous studies have provided valuable insights into AI's applications in nursing, such as patient monitoring, risk prediction, personalized care planning, and decision support systems (9–11, 101). Reviews have also examined ethical considerations, the integration of AI into nursing workflows, and the perspectives of nursing professionals (12–16). While these contributions are significant, a detailed critical evaluation of the current research landscape and the identification of key knowledge gaps remain underexplored.

This review was designed to address this gap through a critical analysis of the current state of knowledge, identifying the challenges and impacts of AI in nursing, and highlighting areas that require further exploration. The originality of this work lies in its focused attention on research gaps within the intersection of nursing and AI. Through synthesizing current knowledge and highlighting areas with insufficient evidence, this review aims to guide future efforts toward the effective and ethical integration of AI into nursing practice. This analysis advances academic discussions while serving as a resource for policymakers, educators, and leaders in nursing as they adapt to the expanding role of AI in healthcare.

## 2 Current applications of AI in nursing

The integration of AI into nursing has driven significant advancements across various aspects of healthcare delivery. This section explores the primary applications of AI in nursing, emphasizing how these technologies are transforming practices, improving patient care, and optimizing operational efficiency.

### 2.1 Clinical decision support systems

AI-powered clinical decision support systems have become essential tools in nursing practice, assisting in clinical judgment and contributing to improved patient outcomes. These systems use advanced analytics to process and interpret large volumes of patient data, including electronic health records, vital signs, and laboratory results (90). By identifying patterns and providing evidence-based recommendations, they support nurses in making more informed care decisions.

One impactful application of these tools is in predicting patient deterioration. AI algorithms have been developed to analyze real-time patient data and predict the risk of adverse events or clinical decline with high accuracy (17). Escobar et al. (18) demonstrated that an AI-based early warning system significantly reduced in-hospital mortality and length of stay, showcasing the potential of these technologies to enable timely interventions and prevent negative outcomes.

AI-driven systems have also demonstrated significant efficacy in optimizing medication management, which is a vital aspect of nursing care. These systems leverage advanced algorithms to analyze patient data, resulting in improved medication adherence, reduced errors, and enhanced therapeutic outcomes. The integration of artificial intelligence into clinical settings not only streamlines the medication process but also supports healthcare professionals in making informed decisions, emphasizing its potential to revolutionize patient care by facilitating personalized medication strategies (19, 20).

### 2.2 Patient monitoring and predictive analytics

AI-powered patient monitoring technologies have revolutionized how nurses track and respond to patients' health conditions. These systems allow for continuous, real-time analysis of extensive patient data, providing unparalleled insights into a patient's status.

This capability is particularly valuable in high-acuity environments, such as intensive care units, where early detection of changes in a patient's condition is essential. Wearable devices integrated with AI algorithms can monitor vital signs, activity levels, and other physiological markers continuously (21, 22). Advanced analytics applied to this data can detect early warning signs and alert nurses, enabling prompt interventions (23). Li et al. (24) demonstrated that an AI-powered wearable system could predict exacerbations in patients with chronic obstructive pulmonary disease, facilitating proactive management strategies that reduced hospital admissions and improved patient outcomes.

Beyond monitoring, AI is also being used to advance predictive analytics in nursing. Sophisticated machine learning models can forecast patient trajectories and resource requirements. Nemati et al. (25) highlighted an AI algorithm capable of predicting the onset of sepsis in ICU patients up to 12 h before clinical recognition, potentially enabling life-saving interventions. In addition, AI algorithms have been used to predict the risk of hospital-acquired infections, allowing nurses to implement targeted preventive measures and improve infection control practices (26–28).

### 2.3 Administrative and workflow optimization

AI applications in nursing extend beyond direct patient care, offering innovative solutions to reduce administrative burdens and improve workflow efficiency. Natural Language Processing technologies, for example, are increasingly used to automate documentation processes (29, 99). These systems can transcribe nurse-patient interactions, generate clinical notes, and populate electronic health records, significantly reducing the time spent on paperwork. Moreover, the use of Natural Language Processing has shown potential to improve the accuracy and completeness of clinical documentation, enhancing overall documentation quality (30).

AI plays a significant role in scheduling and resource allocation, which are critical areas for nursing management. Advanced algorithms have been developed to analyze a variety of data, including staff availability, patient acuity, and historical workload patterns, enabling the creation of optimized schedules that meet the needs of both healthcare professionals and patients. This approach enhances operational efficiency and also contributes to the quality of patient care by minimizing staff stress and improving overall satisfaction. The implementation of AI in shift scheduling reduces the time spent on administrative tasks, allowing nurses to focus more on patient care (31, 32). Furthermore, AI-driven scheduling tools can adapt in real-time to changes in patient needs or staff availability, ensuring that resources are allocated effectively and that patient care remains a top priority (33).

AI also plays a role in scheduling and resource allocation, critical areas for nursing management. Algorithms have been developed to analyze data such as staff availability, patient acuity, and historical workload patterns to create optimized schedules. Arnould et al. (116) reported that an AI-based scheduling system implemented in a large hospital improved staff satisfaction and reduced overtime costs by 12%, demonstrating its effectiveness in workforce management.

Furthermore, AI-powered chatbots and virtual nursing assistants are employed to manage routine patient inquiries and provide health-related information, freeing nurses to focus on more complex tasks (34, 103). These tools can engage with patients, answer frequently asked questions, and offer guidance on self-care, medication adherence, and appointment scheduling, enhancing the accessibility of nursing services while allowing clinicians to prioritize direct patient care (35).

### 2.4 Education and training

AI is also transforming nursing education and training by offering innovative approaches to learning and skill development (96, 97). Virtual reality and augmented reality platforms, enhanced through AI, provide simulated clinical scenarios that enable experiential learning (100, 104).

AI-driven adaptive learning systems tailor educational content to an individual's performance, potentially improving the effectiveness of training (36, 102). Chen et al. (37) observed that nursing students who used an AI-powered adaptive learning platform achieved higher clinical reasoning scores compared to those using traditional methods. In another context, AI has been incorporated into the development of advanced patient simulators that respond realistically to interventions, creating a safe environment for nursing students to practice complex procedures and decision-making (38).

### 2.5 Concluding remarks

The current applications of AI in nursing demonstrate its transformative potential to enhance clinical decision-making, improve patient monitoring, streamline administrative tasks, and revolutionize education and training. These advancements not only optimize nursing workflows but also contribute to better patient outcomes and overall healthcare efficiency. While these applications illustrate the transformative potential of AI in nursing, many remain in the early stages of implementation or are limited to experimental settings. Despite the promising developments presented in this section, the integration of AI into nursing practice is not without challenges.

As AI technologies continue to evolve, their adoption in nursing must address critical barriers, including technical limitations, ethical concerns, data privacy issues, and the need for workforce adaptation. These challenges, which will be explored in detail in Section 3, highlight the importance of a thoughtful and collaborative approach to ensure that the implementation of AI aligns with the core principles of nursing care and fosters trust among healthcare professionals and patients alike.

## 3 Challenges in implementing AI in nursing

The integration of AI into nursing practice, while promising, faces numerous challenges that must be addressed to ensure successful implementation. This section explores the key obstacles encountered in adopting and using AI technologies in nursing.

### 3.1 Technical challenges

One of the primary technical challenges in implementing AI in nursing is the quality and standardization of data. AI algorithms, particularly advanced machine learning models, require large, diverse, and high-quality datasets to function effectively and avoid biases. However, healthcare data often suffers from inconsistencies, incompleteness, and lack of standardization across different systems and institutions (39).

Recent studies demonstrate that variations in documentation practices, terminologies, and data formats across multiple healthcare facilities significantly hindered the development of accurate predictive models for patient outcomes (105). These studies highlight the need for comprehensive data governance frameworks and standardization initiatives to address these issues and enable the effective deployment of AI in nursing.

Interoperability between AI systems and existing healthcare infrastructure presents another significant hurdle (40, 106). Many healthcare facilities use legacy systems that may not be compatible with new AI technologies, necessitating substantial investments in infrastructure upgrades, data integration, and system interoperability (41). A report from the National Academy of Medicine revealed that most healthcare organizations cited interoperability as a major barrier to AI adoption, underscoring the scale of this challenge (National Academy of Medicine, 2018).

Furthermore, the “black box” nature of some AI algorithms, particularly advanced deep learning models, poses challenges for transparency and explainability (42, 107). In healthcare, where decisions can have life-or-death consequences, the inability to fully understand or explain AI-driven decisions is problematic. This lack of transparency can raise concerns about the trustworthiness and reliability of AI systems, potentially hindering their acceptance and adoption by healthcare professionals, including nurses (108). Ongoing research efforts in the field of explainable AI aim to address this challenge and improve the interpretability of AI-powered decision-making processes.

### 3.2 Ethical considerations

The use of AI in nursing raises several ethical concerns. A key issue is the potential for AI systems to perpetuate or even amplify existing biases in healthcare. If the data used to train AI models contains historical biases, the resulting systems may produce unfair or discriminatory outcomes, exacerbating health disparities (43).

Accountability is another critical concern. Determining who is responsible for errors or adverse outcomes involving AI systems remains a complex legal and ethical challenge (44).

Furthermore, the integration of AI into nursing care may challenge the principle of human-centered care. Over-reliance on AI could risk dehumanizing healthcare, potentially diminishing the nurse-patient relationship, which is a cornerstone of nursing practice (45).

### 3.3 Data privacy and security

The integration of AI in nursing practice necessitates the collection, storage, and analysis of vast amounts of sensitive patient data, which raises significant privacy and security concerns. Ensuring compliance with data protection regulations, such as the General Data Protection Regulation in Europe or the Health Insurance Portability and Accountability Act in the United States, while simultaneously leveraging the full potential of AI is a complex challenge that requires careful consideration (46).

One critical issue is the potential for cybersecurity threats targeting AI systems in healthcare (47, 48, 93). A breach in an AI system could not only compromise patient privacy but also lead to erroneous clinical decisions if the system's integrity is compromised (49). This threat is particularly worrying given the life-or-death consequences that can arise from faulty clinical decisions made by AI-powered systems. As a recent study demonstrated, the ransomware attack on a hospital's AI-enabled medical imaging system led to delayed diagnoses and treatment for several patients, potentially contributing to adverse health outcomes (50). Similarly, a report by the Ponemon Institute revealed that healthcare organizations faced an average increase of $7.13 million in data breach costs when AI and IoT technologies were involved, underscoring the substantial financial and reputational risks associated with such incidents (109).

To address these challenges, robust cybersecurity measures are essential, such as implementing strong access controls, employing data encryption techniques, and conducting regular security audits. Also, the development of ethical guidelines and regulatory frameworks specifically tailored to the use of AI in healthcare settings can help ensure the protection of patient data and the integrity of AI-driven clinical decision-making.

### 3.4 Ownership aspects of patient data

As AI continues to integrate into nursing practice, the question of ownership regarding patient data used for AI training becomes increasingly pertinent. The ethical implications of data ownership not only affect patients but also impact the roles and responsibilities of nursing professionals in ensuring that patients' rights are upheld. The Global Patient co-Owned Cloud presents a novel framework for considering patient data ownership, advocating for a shared ownership approach where patients co-own their personal health records alongside healthcare providers (51, 52). This model empowers patients to have greater control over their data, fostering a sense of partnership in their healthcare journey.

In this context, nurses play a critical role as advocates for patients, ensuring that they are informed about how their data is used and the potential benefits and risks associated with AI technologies (53). Promoting transparency helps build trust between patients and healthcare systems, facilitating a more collaborative environment for data sharing. From an ethical standpoint, the co-ownership of personal health records data raises several important questions. Nurses must consider the implications of data privacy, consent, and the potential for misuse of information. It is essential that patients are educated about their rights regarding data ownership and are actively involved in decisions about how their data is used in AI applications.

Furthermore, nurses must navigate the ethical dilemmas that arise when balancing the benefits of AI, such as improved patient outcomes and enhanced care delivery, with the need to protect patient autonomy and confidentiality (54). Establishing comprehensive ethical frameworks tailored to nursing contexts is crucial for guiding nursing professionals in these complex situations. To fully realize the potential of co-ownership models like Global Patient co-Owned Cloud, further research is needed to explore the implications of patient data ownership in AI applications. Investigating the perspectives of nursing professionals on this issue will provide valuable insights into how to navigate ethical challenges and promote patient-centered care in the age of AI. Addressing these ownership aspects allows the nursing profession to better align AI advancements with the needs and rights of patients, ultimately enhancing the quality of care and fostering a more ethical healthcare environment.

### 3.5 Integration with existing healthcare systems

Integrating AI technologies into established healthcare workflows and systems presents significant challenges. Many healthcare professionals, including nurses, may be resistant to adopting new technologies that disrupt familiar routines or require substantial retraining.

The integration of AI systems into established healthcare workflows and procedures often necessitates a substantial redesign of clinical processes. This can be a complex and resource-intensive undertaking, as it involves carefully aligning new AI-powered tools and technologies with existing clinical practices and infrastructures. One key challenge is ensuring that the implementation of AI does not inadvertently disrupt the efficiency and productivity of nursing workflows (8). If the incorporation of AI technologies introduces additional steps, creates unnecessary complications, or fails to seamlessly integrate with current systems, it can lead to frustration, decreased productivity, and resistance from nursing staff (55, 56).

To address this, it is importance taking a user-centric approach to AI integration, where the needs and workflows of nurses are thoroughly mapped and prioritized during the design and implementation process (110, 111). This may involve conducting in-depth analyses of current nursing practices, identifying pain points and bottlenecks, and designing AI solutions that augment and enhance these existing processes rather than create new ones.

Furthermore, the successful integration of AI often requires substantial investments in infrastructure upgrades, staff training, and organizational change management (57). Hospitals and healthcare systems must be willing to commit the necessary resources to ensure that the introduction of AI tools aligns with and optimizes, rather than disrupts, nursing workflows (112). Only through this holistic and strategic approach can the full potential of AI be realized in enhancing the efficiency and productivity of nursing practice.

### 3.6 Workforce adaptation and training

The introduction of AI in nursing necessitates significant changes in education and professional development. Current nursing curricula may not adequately prepare students to work with AI technologies, creating a skills gap in the workforce (8).

Continuous training and upskilling are critical for the existing nursing workforce to effectively use and interpret AI tools, which requires investments in education programs and a cultural shift within healthcare organizations to prioritize technological competence alongside traditional nursing skills (58).

There are also concerns about the potential impact of AI on nursing roles and employment. While AI is generally viewed as a tool to augment nursing practice, fears of automation replacing certain tasks may lead to job displacement or redefinition of roles (59).

### 3.7 Concluding remarks

Despite its potential to revolutionize nursing practice, the implementation of AI is accompanied by multifaceted challenges. Addressing these obstacles requires collaboration among healthcare providers, policymakers, technology developers, and educators. Ensuring that AI adoption aligns with the core values of nursing care is paramount.

The complexity of these challenges highlights the need for further research and interdisciplinary efforts to identify solutions. In the next section, we will explore the current research gaps in this field, emphasizing areas where additional investigation is needed to overcome these barriers and fully realize the transformative potential of AI in nursing.

## 4 Research gaps and future directions

As AI continues to integrate into nursing, several significant gaps in knowledge and application remain. Addressing these gaps is essential to refine AI technologies for nursing practice, ensuring they align with professional standards, improve patient care, and maintain ethical integrity. This section outlines key areas requiring further exploration and proposes directions for advancing the field.

### 4.1 Limitations in current AI applications

Although AI has demonstrated potential in various nursing contexts, there is a lack of large-scale, longitudinal studies assessing its efficacy and safety in real-world clinical environments. Much of the existing evidence is derived from small-scale pilot projects or conceptual demonstrations, which limits the generalizability of findings and the ability to assess long-term impacts (60).

To address this limitation, future work should focus on rigorous, multi-site clinical trials that evaluate the sustained effects of AI on patient outcomes, nursing efficiency, and healthcare costs (61). AI-enabled decision support systems could be systematically assessed for their role in chronic disease management, where they may assist nurses in analyzing patient data to recommend tailored care strategies. Similarly, workflow optimization tools, such as automated scheduling or inventory management systems, should undergo evaluation to determine their ability to reduce administrative burdens and increase productivity (62, 63).

Comprehensive studies of this nature would provide the empirical foundation needed to assess the practicality, safety, and economic viability of AI technologies in nursing. Such evidence is critical for guiding policy decisions and facilitating the broader adoption of AI across healthcare systems (64).

### 4.2 Underexplored areas in nursing-specific AI

While much of the current AI research in healthcare has concentrated on medical diagnosis and treatment planning (26), a significant gap exists in exploring AI applications specifically tailored to the unique aspects of nursing practice. Nursing encompasses a wide range of specialized competencies, including patient education, care coordination, holistic patient assessment, and the management of complex clinical scenarios.

Future investigations should prioritize the development of AI-powered tools and technologies designed to enhance and augment these core nursing skills. AI-driven communication assistants could enable nurses to engage more effectively with patients, facilitating personalized education on disease management, medication adherence, and lifestyle modifications (65). Similarly, AI-enabled care planning systems could assist nurses in formulating and continuously optimizing individualized care strategies, drawing insights from extensive patient data, evidence-based guidelines, and best practices (3).

Furthermore, AI presents substantial potential for enhancing nursing interventions in challenging areas such as wound care management (66). By leveraging natural language processing and predictive analytics, AI-powered systems could aid nurses in conducting comprehensive wound assessments, tracking healing progress, and recommending tailored treatment plans. This approach could lead to improved patient outcomes, reduced complications, and more efficient utilization of nursing resources.

Exploring these nursing-specific AI applications can yield valuable insights and accelerate the integration of transformative technologies into the nursing profession. Addressing the unique needs and workflows of nurses will ensure that the development and implementation of AI solutions align with and enhance the core objectives of nursing care, ultimately improving patient outcomes and the overall efficiency of healthcare delivery.

### 4.3 Interdisciplinary collaboration

Developing effective AI solutions for nursing necessitates close collaboration among diverse stakeholders, including nurses, computer scientists, ethicists, and other healthcare professionals. Currently, a lack of structured frameworks hampers such interdisciplinary cooperation (67).

Addressing this gap is vital for the successful integration of AI into nursing practice. It is essential to develop and evaluate robust models of interdisciplinary teamwork in the context of AI development for nursing (11, 68). Exploring innovative approaches to integrate nursing expertise into the AI design process will enable nurses to contribute their domain-specific knowledge and clinical perspectives.

Investigation into methods to enhance knowledge transfer between technical and clinical teams is equally important. This may involve creating shared communication frameworks, fostering cross-training initiatives, and establishing collaborative workflows that bridge the technical and practical aspects of AI implementation. Facilitating the exchange of knowledge and expertise across disciplines will ensure that AI solutions for nursing align with the unique needs, workflows, and ethical considerations of the profession.

The establishment of structured interdisciplinary collaboration models (69, 113) is essential for bridging the gap between the promise of AI and its practical application in nursing care. This collaborative approach will enable the creation of AI technologies that enhance nursing efficiency, improve patient outcomes, and align with the core values and objectives of the nursing profession.

### 4.4 Development of ethical frameworks

The integration of AI into nursing practice raises complex ethical questions, particularly regarding patient autonomy, data privacy, and the preservation of the therapeutic nurse-patient relationship. While general ethical guidelines for AI in healthcare exist, there is a need for frameworks tailored specifically to nursing (70).

Future studies should focus on creating detailed ethical models that address the unique challenges nurses face when working with AI systems (71). These models should provide guidance on balancing the efficiency of AI tools with the need for personalized, compassionate care. They should also address issues such as equitable access to AI-enabled interventions and the importance of obtaining informed patient consent when AI technologies are used in care delivery.

Clear ethical guidelines empower nursing professionals to navigate the complexities of AI integration while maintaining their commitment to patient-centered care and professional accountability.

### 4.5 Long-term impact assessments

The long-term effects of AI on nursing practice, workforce dynamics, and patient care remain poorly understood. Comprehensive longitudinal studies are needed to evaluate how AI technologies influence nursing roles, job satisfaction, and the quality of care over extended periods (114).

Such studies should also investigate potential unintended consequences of AI adoption, including changes in clinical reasoning skills, alterations in nurse-patient interactions, and shifts in nursing education and professional development. Additionally, the societal implications of AI-driven changes in nursing, including their impact on healthcare access and equity, warrant further investigation.

Addressing these questions through long-term studies will provide valuable insights into the broader implications of AI for the nursing profession and healthcare systems.

### 4.6 Education and training

There is a growing need to incorporate AI-related content into nursing education to prepare practitioners for the evolving healthcare environment. Current nursing curricula often lack sufficient coverage of AI technologies and their implications for clinical practice (8).

Efforts should focus on developing and implementing educational programs that equip nurses with the knowledge and skills to work effectively in AI-integrated settings. This could include the use of AI-enhanced simulation tools to provide hands-on training in managing AI-enabled systems, as well as courses on the ethical and practical considerations of AI in nursing care.

Strengthening AI literacy within the nursing profession supports the safe and effective adoption of AI technologies in clinical practice.

### 4.7 Explainability and transparency

The “black box” characteristic of numerous AI algorithms presents significant barriers to their acceptance and implementation within nursing practice. There is an imperative for research focused on the development of more transparent and interpretable AI models that can be readily comprehended and trusted by both nursing professionals and patients (72).

Enhancing the explainability and interpretability of AI systems should be a primary focus for researchers in this domain. AI systems capable of providing clear and comprehensible elucidations of their decision-making processes will cultivate increased trust and confidence among nursing practitioners, thereby facilitating the seamless integration of these technologies into nursing workflows (4).

Studies should investigate the establishment of robust mechanisms for AI oversight and accountability (73), ensuring that nurses and patients have a comprehensive understanding of the decision-making processes and can hold AI systems accountable for their recommendations and actions (74). Addressing the critical issues of explainability and trust will enable the nursing profession to fully harness the potential of AI-driven solutions while upholding the core values of patient-centered care and ethical practice.

### 4.8 Cultural and contextual adaptability

While much of the existing AI research in healthcare has predominantly concentrated on high-resource settings, a significant gap persists in understanding how these transformative technologies can be effectively adapted to diverse cultural contexts and resource-limited environments (75).

Future investigations must prioritize innovative approaches to tailoring AI solutions to address the unique needs of various healthcare systems and cultural contexts (76, 77, 94). This may involve examining the development of low-cost, AI-powered nursing care solutions specifically designed for underserved communities in developing countries, where access to advanced medical technologies is often restricted (78).

A strong emphasis should also be placed on studying the cultural acceptability and sustainability of AI technologies across a broad spectrum of global settings (79), which includes analyzing how diverse cultural norms, values, and beliefs influence the perception and integration of AI-driven nursing interventions. A better understanding of these contextual factors will enable the design of AI solutions that align more closely with the specific needs and preferences of diverse patient populations.

Furthermore, future studies should explore strategies for adapting AI systems to function effectively in resource-constrained environments, where challenges such as limited infrastructure, intermittent power supply, and a scarcity of specialized personnel may arise (75). Innovative methodologies, including edge computing, distributed AI, and low-power hardware, could be pivotal in facilitating the deployment of AI-enhanced nursing care in these contexts.

Prioritizing the development of culturally and contextually adaptable AI solutions for nursing will help ensure that the transformative potential of these technologies is realized across a wide array of global healthcare settings, ultimately enhancing access to high-quality, patient-centered nursing care for all.

### 4.9 Concluding remarks

The exploration of research gaps and future directions highlights the critical areas requiring attention to fully unlock the potential of AI in nursing. Addressing limitations in current applications, fostering interdisciplinary collaboration, developing ethical frameworks, and ensuring cultural adaptability are essential steps toward creating AI solutions that meet the unique needs of nursing practice. Advancing AI literacy, enhancing explainability, and conducting long-term impact studies will establish a strong foundation for the sustainable and ethical integration of AI technologies into nursing care.

As the field progresses, bridging these research gaps through robust studies and collaborative efforts remains imperative. The next section will focus on the potential developments and opportunities AI presents for nursing practice. The discussion will emphasize how tackling the identified challenges can lead to innovations that enhance personalized care, improve efficiency, and support education and workforce development. Emerging advancements and interdisciplinary efforts will be explored as key drivers shaping the future of nursing, ensuring AI technologies remain effective and aligned with the core values of patient-centered care and ethical practice.

## 5 Potential developments and opportunities

As the gaps outlined in the previous section are addressed, numerous opportunities for advancements in AI within nursing practice become apparent. This section highlights key areas of potential development that could transform nursing care and improve patient outcomes.

### 5.1 Personalized care and precision nursing

The integration of AI with genomics, proteomics, and other -omics data offers significant potential for advancing personalized nursing care. AI algorithms can process extensive datasets to create individualized care plans that consider genetic predispositions, lifestyle factors, and environmental influences (80).

One promising innovation involves the creation of “digital twins”—virtual representations of individual patients that enable simulations of care strategies before real-world implementation (81). These models allow nurses to test various interventions and predict outcomes, refining their approach to deliver highly tailored care.

Such advancements could lead to a paradigm shift in healthcare delivery, enabling nurses to provide interventions that are specifically designed for each patient's unique circumstances. These tools have the potential to improve clinical outcomes, enhance patient satisfaction, and elevate the overall quality of care.

### 5.2 Advanced robotics in nursing

AI-powered robotics represents a transformative opportunity for nursing. Future studies may result in robotic systems capable of performing complex tasks such as patient lifting, medication administration, and wound care, which could reduce the physical demands on nursing staff and improve workflow efficiency (34, 58).

Socially assistive robots equipped with natural language processing and emotional recognition capabilities could also play an important role in patient care (95). These robots might provide companionship and basic support for elderly or chronically ill patients, addressing issues such as loneliness while enabling nurses to focus on more specialized aspects of care (82, 83).

The integration of robotics into nursing has the potential to complement, rather than replace, human nurses. Combining human expertise with robotic precision could lead to more efficient, personalized, and compassionate care delivery across diverse healthcare settings.

### 5.3 AI-driven nursing education

AI technologies are set to revolutionize nursing education through personalized and adaptive learning systems. These systems can adjust content delivery, pacing, and teaching strategies to meet the specific needs and learning styles of individual students (37, 84). Such customization ensures that nursing students receive targeted support to excel academically and clinically.

Virtual and augmented reality simulations powered by AI offer additional opportunities for students to gain hands-on experience in a controlled environment (85, 86). These simulations replicate the complexities of real-world healthcare scenarios, allowing students to practice skills and decision-making without risk to actual patients (115).

AI can also enhance faculty insights into student performance. Analytical tools can identify areas where students may need additional support, enabling educators to design tailored interventions that foster academic success and professional readiness (87).

### 5.4 AI-enabled continuous learning

AI systems present significant opportunities for continuous improvement in nursing practice using data-driven insights. Through analyzing patterns in patient outcomes, nursing interventions, and workflow efficiency, these systems can identify best practices and highlight areas that require enhancement (60). This analytical capability allows nurses to understand which interventions yield the best results, ultimately leading to improved patient care.

The feedback generated from these analyses is crucial for developing adaptive health systems that can evolve in response to changing patient needs and dynamic healthcare environments (12, 88). Such systems facilitate ongoing refinement of care delivery, ensuring that nursing practices remain current and effective in addressing emerging challenges.

AI-enabled continuous learning fosters a culture of innovation within nursing teams. Nurses gain access to real-time data and insights, empowering them to make informed decisions, engage in evidence-based practice, and collaborate more effectively with interdisciplinary teams. This collaborative approach enhances individual nursing practice and significantly contributes to the overall quality of care provided within healthcare settings.

As AI technologies advance, the potential for continuous learning in nursing will expand, leading to even greater adaptability and responsiveness. Embracing these innovations ensures that the nursing profession remains at the forefront of healthcare delivery, equipped to meet the evolving needs of patients and communities. This commitment to continuous improvement enhances patient outcomes and strengthens the nursing workforce, fostering an environment where professional growth and development thrive.

### 5.5 Predictive analytics for population health

Advances in AI will revolutionize population health management through the use of predictive analytics. On leveraging sophisticated algorithms, AI-powered tools identify high-risk individuals or communities, facilitating early interventions and more efficient allocation of healthcare resources (89). Such a proactive approach enhances patient outcomes while optimizing the use of healthcare services.

Future systems could integrate diverse data sources, including electronic health records, wearable devices, and environmental sensors. This integration would provide real-time insights into health trends and emerging risks, enabling nurses to monitor population health more effectively. Analyzing data from wearable devices enables nurses to track vital signs and activity levels, allowing them to identify patients who may be at risk for chronic conditions before symptoms arise.

With these capabilities, nurses can implement proactive care strategies tailored to specific community needs, which might include developing targeted outreach programs for high-risk populations or delivering personalized health education initiatives that promote preventive care and healthy lifestyle choices. In addition, predictive analytics could guide resource allocation by identifying areas with the greatest need for healthcare services, ensuring that interventions are both timely and effective.

### 5.6 Enhanced decision support systems

Next-generation clinical decision support systems, powered by advanced AI algorithms, have the potential to provide nurses with refined and context-aware recommendations. These systems could integrate not only clinical data but also psychosocial factors, patient preferences, and the latest research findings to guide nursing interventions (90).

The incorporation of these AI-driven decision support tools would offer nurses valuable insights tailored to the unique needs and circumstances of each patient (91). On considering a broader range of variables (including social, emotional, and lifestyle factors), these systems could assist nurses in developing more personalized and effective care plans (92).

Future studies may include AI assistants capable of natural language interaction, enabling nurses to query complex clinical scenarios and receive evidence-based guidance in real-time. This interactive capability would allow nurses to quickly access relevant information and recommendations, empowering them to make informed decisions and deliver optimal care to their patients.

### 5.7 Ethical AI frameworks

The establishment of ethical frameworks tailored to AI in nursing is essential for aligning technological advancements with the profession's core values. These frameworks guide nurses in navigating ethical dilemmas related to data privacy, patient autonomy, and equitable access to AI-driven care.

A primary consideration is the protection of patient data. Ethical guidelines must prioritize data privacy and security, establishing clear protocols for data collection, storage, and sharing. Nurses should be trained in best practices for handling patient information to maintain trust and confidentiality, which are fundamental to the nurse-patient relationship.

Respecting patient autonomy is equally relevant. Patients must be fully informed about how AI technologies will be used in their care, including clear explanations of AI-driven recommendations. This ensures that patients can make informed choices about their treatment options, reinforcing the ethical commitment to empower individuals in their healthcare decisions.

Equitable access to AI technologies is another significant concern. Ethical frameworks should advocate for policies that promote inclusivity, ensuring that the benefits of AI-driven care are accessible to all patients, particularly underserved populations.

Finally, ethical frameworks must encourage a balance between leveraging AI's benefits and preserving the human connection central to nursing. While AI enhances efficiency, compassion and personal interaction must remain priorities. Continuous evaluation of AI's impact on nursing practice will be vital for refining these frameworks, ensuring they effectively guide ethical decision-making in an evolving technological landscape. Ultimately, robust ethical frameworks will enhance patient care while upholding the ethical standards of the nursing profession.

### 5.8 Concluding remarks

The potential developments outlined in this section represent transformative opportunities for nursing. The integration of AI into personalized care, robotics, education, and decision-making has the capacity to enhance efficiency, improve outcomes, and elevate the quality of patient care. However, these advancements must be pursued with careful consideration of ethical implications, ensuring that technology serves to augment and empower nursing professionals rather than replace the critical human elements of care.

## 6 Conclusion

This review provided a comprehensive analysis of the challenges and impacts of AI in nursing, emphasizing both its substantial potential and the significant efforts required to realize it fully. The evaluation of current applications demonstrates that AI is already making meaningful contributions to nursing practice, though much remains to be explored and developed.

Key priorities for advancing AI in nursing include the development of nursing-specific AI solutions, the establishment of robust ethical frameworks, workforce preparation, long-term impact studies, and the promotion of interdisciplinary collaboration. It is essential for the nursing profession to play an active role in shaping AI's development to ensure that emerging solutions align with nursing values and enhance patient care. Maintaining a balanced perspective on AI's benefits and limitations will be critical to leveraging its potential effectively.

This review also identified critical challenges that must be addressed, including technical limitations, ethical considerations, data privacy concerns, and the need for workforce adaptation. While these challenges are significant, they are not insurmountable and require thoughtful, evidence-based approaches. Furthermore, research gaps were highlighted, particularly the need for nursing-specific AI applications, comprehensive ethical frameworks, and longitudinal studies to evaluate the long-term impacts of AI on patient care and nursing practice.

The potential developments discussed in this review illustrate a future where AI could profoundly enhance nursing capabilities. However, achieving this vision will require sustained effort, collaboration across disciplines, and a commitment to addressing the identified challenges.
